# *Lentinula edodes* Cultured Extract Intake at Puberty Mitigates Inflammatory Signals at the Mammary Glands by the Involvement of Epigenetic Mechanisms in BALB/c Mice

**DOI:** 10.1155/tbj/2122220

**Published:** 2026-03-31

**Authors:** Hamed Yasavoli-Sharahi, Roghayeh Shahbazi, Nawal Alsadi, Nasim Bondar Sahebi, Cyrille Cuenin, Vincent Cahais, Felicia Fei-Lei Chung, Zdenko Herceg, Chantal Matar

**Affiliations:** ^1^ Department of Cellular and Molecular Medicine, Faculty of Medicine, University of Ottawa, Ottawa, Ontario, Canada, uottawa.ca; ^2^ Latner Thoracic Surgery Laboratories, Princess Margaret Cancer Centre, University Health Network, Toronto, Ontario, Canada, uhn.ca; ^3^ Department of Computer Science, University of Toronto Mississauga, Mississauga, Ontario, Canada, utoronto.ca; ^4^ Epigenomics and Mechanisms Branch, WHO International Agency for Research on Cancer (IARC), Lyon, France; ^5^ Department of Medical Sciences, School of Medical and Life Sciences, Sunway University, Petaling Jaya, Selangor, Malaysia, sunway.edu.my; ^6^ School of Nutrition Sciences, Faculty of Health Sciences, University of Ottawa, Ottawa, Ontario, Canada, uottawa.ca

**Keywords:** DNA methylation, LPS, mammary glands’ immunity, microbiome, miRNAs, prebiotics, proinflammatory cytokines

## Abstract

Exposure to immune stress or lipopolysaccharide (LPS) during critical developmental stages like puberty may lead to gut microbiome dysbiosis and epigenetic dysregulation in mammary glands, affecting gene expression and potentially elevating breast cancer susceptibility in adulthood. Although LPS’s adverse impacts on intestinal and brain functions are well‐documented, its effects on mammary glands remain underexplored. Using an immunocompetent BALB/c mouse model, we administered an acute LPS dose (1.5 mg/kg body weight) during puberty. The study evaluated the long‐term consequences of LPS exposure alone and combined with AHCC (*Lentinula edodes* cultured extract, 2 g/kg body weight/day) on DNA methylation patterns, cytokine profiles, and microRNA expression in mammary glands at 9 weeks of age. Analyses included DNA methylation sequencing, multiplex immunoassays, quantitative PCR, and image processing. Pubertal LPS exposure produced persistent molecular dysregulation in mammary glands, including differential DNA methylation (> 5% change vs. control; FDR‐adjusted *p* < 0.05), elevated inflammatory mediators, and altered microRNA expression. Differentially methylated regions were enriched in regulatory features, with decreased methylation at transcription start sites, promoters, and 5′ UTRs of genes implicated in mammary development and oncogenic signaling (including *Vav3, Pdgfa, Pdgfc, Jag2, Hras, Ksr1, Il2rb, Il17b,* and *Il17rb*) in the LPS group, whereas the AHCC + LPS group exhibited a shift toward hypermethylation at these loci (approximately 5%–10% decrease). Inflammatory profiling showed increased IL‐17A/F (∼2‐fold vs. control; *p* < 0.05), while microRNA analyses indicated reduced let‐7a/c (∼30% vs. control; *p* < 0.05). Notably, miR‐130a and miR‐34a increased ∼1.5‐fold across all treatment groups relative to control. Pubertal LPS exposure induces enduring epigenetic and inflammatory changes in mammary glands that may heighten breast cancer risk. AHCC’s mitigating role indicates potential for dietary interventions to counteract these effects.

## 1. Introduction

The commensal gut microbiota significantly impacts human health, particularly the development and function of the immune system [1, 2]. The intricate balance of the gut microbiome can be disrupted by various factors, including diet, medication, and obesity, leading to long‐lasting metabolic and immune consequences, particularly in cases of childhood obesity and compromised immunity [3, 4].

Dysbiosis of the gut microbiome leads to an increased presence of circulating lipopolysaccharide (LPS), which triggers an immune response [5]. Changes in gut permeability and alterations in the gut microbiota, caused by factors like diet or immune stressors, coincide with changes in gut function and contribute to dysfunctional immune responses characterized by systemic inflammation [6, 7]. The inflammation‐related modifications in the gut microbiome further compromise the integrity of the intestinal barrier. LPS, being a potent inducer of inflammatory responses, raises the possibility of an association between LPS and susceptibility to cancer. The circulating LPS or the increase in LPS load spreads to target tissues, imposing a burden not only on the gut but also on distant organs, including mammary glands [8].

The inflammatory response induced by LPS triggers events that lead to the modulation of the Th17 signaling pathway [9]. Within the gut‐associated lymphoid tissue, LPS reduces the frequency of regulatory T cells (Tregs) while promoting the presence of Th17 cells. Th17 cells, essential components of the mucosal‐associated lymphoid tissue, elicit an inflammatory reaction when encountering pathogens or LPS by releasing IL‐17 and IL‐23 [10, 11]. Activation of Th17 cells drives cellular responses, including the secretion of IL‐1β, IL‐6, and IL‐10 and epigenetic changes in gene expression [12]. These effects can impact various cell types, including cells in mammary glands. Importantly, activated Th17 cells possess the ability to migrate to and home in on lymph nodes situated in distant sites, such as breast tissue [13].

Puberty serves as a critical window of susceptibility for epigenetic reprogramming in the mammary gland, driven by rapid epithelial cell proliferation, differentiation, and branching morphogenesis, which heighten vulnerability to environmental stressors [14]. Inflammatory stimuli, such as LPS, can exacerbate this by inducing epigenetic modifications like DNA methylation changes and microRNA dysregulation, leading to persistent gene expression alterations that may elevate breast cancer risk. Such inflammation‐driven mechanisms alter gene expression patterns at the gut and distant sites, including mammary glands [15, 16]. Extensive research has highlighted the impact of inflammation on DNA methylation and microRNA expression [17, 18]. DNA methylation plays a critical role in the differentiation of mammary glands by influencing critical transcription factors, including HOXA1, TCF7L1, and GATA3 [19]. Additionally, microRNAs (miRNAs) also contribute significantly to the differentiation of terminal end buds. For instance, specific miRNAs, such as miR‐184, exhibit pronounced upregulation during the differentiation of pubertal terminal end buds [20].

Prebiotics are essential for the maintenance and regulation of the gut microbial ecosystem [21]. They not only contribute to reducing inflammation throughout the body but also counteract the detrimental effects of LPS when used as part of dietary manipulation with probiotics [22]. The beneficial effects of prebiotics primarily target the gut microbiota and have been demonstrated to exert influence on gene expression at the epigenetic level [23]. Remarkably, the gut microbiota possesses the capability to impact the Th17 signaling pathway, thereby affecting lymphocytes in distant sites [24]. Furthermore, prebiotics interact with gut and immune cells, playing a regulatory role in modulating inflammatory pathways. This regulation is achieved through the modulation of pattern‐recognition receptors (PRRs), specifically Toll‐like receptors, as well as key signaling pathways such as NF‐kB [25].

The mammary gland undergoes a unique developmental process, starting during the fetal stage, temporarily pausing after birth, and resuming in response to estrogen during puberty [26]. The regulation of mammary gland morphogenesis, particularly the formation of terminal end buds and secondary branch points, involves various mechanisms such as DNA methylation and miRNAs [19, 20]. At the onset of puberty, inflammation triggers the activation of crown‐like structure macrophages, leading to the release of inflammatory factors that not only disturb the gene expression patterns of mammary stem cells but also impact adipokine expression within the fat pad surrounding mammary glands [27]. Additionally, pubertal exposure to LPS has been shown to elicit an immune response and influence brain function [28], yet its lasting effect on mammary glands remains understudied. The intake of prebiotics may potentially mitigate the inflammatory effect of LPS, although the impact of prebiotic intake in regulating the prolonged effects of inflammation on mammary glands has not been thoroughly investigated. Therefore, the objective of this study is to examine the effects of pubertal LPS exposure on mammary glands and evaluate whether dietary intervention during this critical period can reduce LPS‐induced dysbiosis and rectify the enduring effects of LPS on mammary gland function.

In pursuit of our research goals, we employed a mouse model that induced inflammation during puberty through the administration of LPS. To explore potential interventions, we provided the mice with a prebiotic compound called *Lentinula Edodes* cultured extract (AHCC), known to possess potent immunomodulatory properties by inhibiting the activation of toll‐like receptor 4 [29]. Within the scope of this study, we aimed to delve into the intricate DNA methylation profiling of mammary glands, assess the level of cytokines, and investigate the expression pattern of microRNAs. We aimed to uncover valuable insights into the potential impact of inflammation and the efficacy of AHCC in modulating epigenetic factors, shedding light on new avenues for therapeutic intervention in mammary gland‐related conditions.

## 2. Material and Method

### 2.1. Animals

Female BALB/c mice, aged three weeks and weighing between 13 and 17 g, obtained from Charles River (Montreal, QC), were utilized in this study. The mice were housed in plastic cages with three mice per cage, providing a controlled environment with a 12 h light/dark cycle and maintaining a temperature of 22°C ± 2°C and humidity at 55% ± 2%. Throughout the study, all groups of mice were provided with a conventional balanced diet without any restrictions. The treatment and maintenance of mice followed the guidelines outlined by the Canadian Council on Animal Care. The Animal Care Committee of the University of Ottawa approved the experimental protocol (HSe‐3191).

### 2.2. Study Design

The experiment was conducted to investigate the long‐term impact of pubertal exposure to LPS and prebiotic intake on the gut microbiota and immune system. A total of 36 mice were utilized for this experiment. Puberty onset was determined by observing the first occurrence of a pubertal event, specifically vaginal opening. The mice were divided into two groups consisting of 18 mice each: the prebiotic group received AHCC (2 g/kg BW/day) in their drinking water, while the control group received regular drinking water for one week prior to puberty. This dosage was chosen as it corresponds to the manufacturer’s recommended human‐equivalent amount, as validated by prior studies [30, 31]. AHCC was administered in drinking water starting 1 week prior to LPS injection and continued for 1 week afterward. This intervention window was chosen based on the hypothesis that AHCC could prime the host immune system and modulate gut microbiota composition before the inflammatory insult, consistent with previous findings that prebiotic compounds exert immunomodulatory effects via gut microbiota remodeling [32]. Upon reaching puberty at 5 weeks of age, half of the mice in each group were injected with a single intraperitoneal injection of LPS at a dose of 1.5 mg/kg body weight, a dose known to induce acute systemic inflammation and gut dysbiosis with lasting physiological effects, even after a single administration [33]. The remaining half received an injection of sterile PBS. The LPS solution was prepared by dissolving LPS from *Escherichia coli* O26:B6 in sterile PBS at a concentration of 0.2 mg/mL. Thus, four experimental groups were formed after the LPS/PBS injection, with nine mice in each group: (1) the control group receiving regular drinking water and PBS injection, (2) the LPS group receiving regular drinking water and LPS injection, (3) the prebiotic group receiving AHCC in drinking water and PBS injection, and (4) prebiotic + LPS group receiving AHCC in drinking water and LPS injection. The nutritional intervention continued for 1 week after injection, followed by a standard diet until early adulthood. At 9 weeks of age, the mice were sacrificed, and mammary gland tissues were collected from all mice (*n* = 9 per group) to examine the enduring effects of the treatment on the immune system (Supporting File 1, Figure S1). The AHCC used in the experiment was provided by Amino Up Co., Ltd., based in Sapporo, Japan.

### 2.3. DNA Extraction and Mouse Methylation BeadChip Array

A segment of 4th inguinal mammary glands from mice weighing 40–50 mg was subjected to homogenization using an electrical homogenizer (Bead Mill 24, Fisher Scientific, Canada). The homogenization was performed in tubes containing 500 μL of cell lysis buffer and 1.5 μL of proteinase K. The lymph node present in the mammary tissue was not removed prior to homogenization. Tissue DNA extraction was carried out using the Gentra PureGene Tissue Kit (33g) (Qiagen, Germany) according to the manufacturer’s instructions. The extracted DNA was subsequently diluted with DNA rehydration solution, provided with the kit, to a final concentration of 20 ng/μL and stored at −20°C. DNA quality was assessed using a Qubit 4 instrument (Thermo Fisher Scientific, Canada) for quantification via fluorescence‐based measurement. Acceptable thresholds included a minimum of 500–600 ng total DNA at a concentration of 20 ng/μL, ensuring purity and avoiding overestimation associated with spectrophotometric methods like NanoDrop. DNA methylation analyses were conducted at the International Agency for Research on Cancer (IARC) in Lyon, France, as previously described [34]. Briefly, 500 ng of extracted DNA was subjected to bisulfite conversion using the EZ DNA Methylation Kit (Zymo Research, Irvine, CA, USA) and was subsequently analyzed using the Infinium Mouse Methylation BeadChip (Illumina Inc., San Diego, CA, USA).

### 2.4. DNA Methylation Data Pre‐Processing, Normalization, and Analysis

DNA methylation analysis was performed using specialized bioinformatics pipeline (detailed in the DNA Methylation Data Pre‐Processing, Normalization, and Analysis section) developed in‐house by the Epigenomics and Mechanisms Branch at the International Agency for Research on Cancer (available at https://github.com/IARCbioinfo/methylkey), which utilizes R packages such as methylkey (v1), SeSAMe (v1.15.1), GenomicFeatures (v1.50.3), and minfi (v1.44.0). Briefly, IDAT files were pre‐processed and normalized using the SeSAMe R package, with robust linear regression applied to identify differentially methylated probes (thresholds: average intergroup difference > |3%| and FDR‐adjusted *p* value < 0.05). Differentially methylated regions (DMRs) were detected using the DMRcate (v2.11.0) package (thresholds: FDR‐adjusted *p* value < 0.05 and |max methylation difference| > 5%). Multiple testing correction was inherently handled via FDR adjustment within these tools. KEGG pathway enrichment analyses were performed using the Enrichr (v3.0) package to identify biological processes associated with DMRs.

### 2.5. Multiplex Luminex Immunoassay

The concentrations of interleukins IL‐1β, IL‐6, IL‐10, IL‐17a, IL‐17f, and IL‐23 were determined using a multiplex Luminex immunoassay (Mouse Th17 Magnetic Bead Panel, catalog number MTH17MAG‐47K, Millipore Sigma, Burlington, Massachusetts, USA). Detection limits (minimum concentrations of the standard curve range) were 15 pg/mL for IL‐1β, 7.8 pg/mL for IL‐6, 20 pg/mL for IL‐10, 39 pg/mL for IL‐17A, 10 pg/mL for IL‐17F, and 342 pg/mL for IL‐23. Intra‐assay variability was < 10% CV and interassay variability was < 15% CV. The extracted proteins from mammary gland samples were diluted with assay buffer solution at a 1:2 ratio. Mouse cytokine standards were prepared at concentrations of 3.2, 12, 80, 400, 2000, and 10,000 pg/mL according to the protocol. Cytokine bead solutions were added to each well of the plate containing the standards, samples, and controls. The plate was then covered and incubated on a plate shaker at 2°C–8°C overnight. Following incubation, two washes were performed, and detection antibodies were added to the wells. The plate was further incubated on a plate shaker at room temperature for 1 h. Streptavidin‐phycoerythrin was subsequently added to each well and incubated at room temperature for 30 min. After another wash, the beads were resuspended in drive fluid and the cytokine concentrations were measured using a MAGPIX system.

### 2.6. Real‐Time Quantitative Reverse Transcription PCR (RT‐qPCR)

A small section of mammary gland tissue was preserved in RNAlater stabilization solution (Invitrogen, USA) at 4°C for 24 h and subsequently stored at −80°C. Total RNA was extracted from the samples using the miRNeasy Mini Kit (Qiagen, Toronto, Canada). The concentration and purity of the extracted RNA were determined using NanoDrop 2000 (Thermo Fisher Scientific, Waltham, MA, USA). For assessing the expression of specific miRNAs, a reverse transcription reaction was performed to synthesize cDNA using the miRCURY LNA RT Kit (Qiagen, USA). The expression levels of microRNA let‐7a, let‐7c, miR‐34, miR‐130a, miR‐146a, miR‐184, miR‐200c, miR‐211, and miR‐219 were measured by RT‐qPCR using the specific miRCURY LNA miRNA PCR assay primers and the miRCURY LNA SYBR Green PCR Kit (Qiagen, Toronto, Canada) on a CFX384 Real‐Time PCR Detection System (Bio‐Rad Laboratories, Hercules, CA, USA). The expression of miRNAs was normalized to the reference gene SNORD65 (mmu) using the miRCURY LNA miRNA PCR assay. The relative expression levels of miRNAs were calculated using the ΔΔCT method.

### 2.7. Whole Mount Staining of Mammary Glands

The fourth inguinal mammary gland was dissected and immediately placed on a glass slide. Subsequently, the slide was immersed in Carnoy’s fixative within a chemical fume hood for 24 h. Following this, the mammary glands were hydrated in 70% ethanol for 15 min and then rinsed in distilled water. The slides were then transferred to a carmine alum solution for storage for 24 h. The dehydration process of the mammary glands involved sequentially transferring the slides into ethanol solutions of increasing concentration from 70% to 100%, followed by an overnight submersion in xylene (Thermo Scientific, Waltham, MA, USA) [35].

### 2.8. Image Processing

**FIGURE 1:**
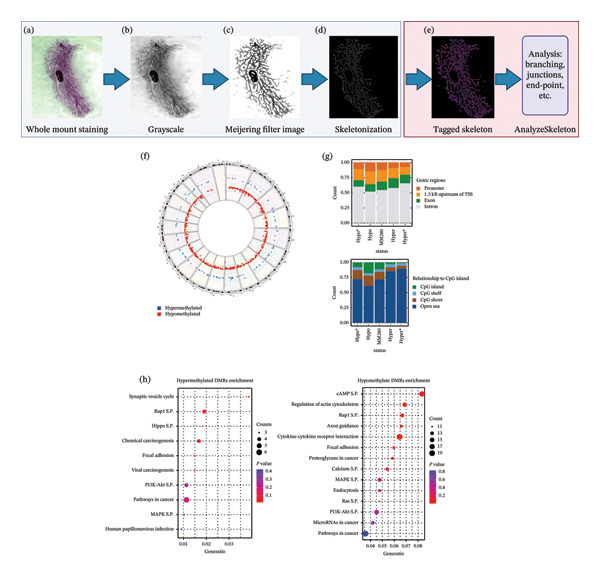
Image processing procedure flowchart and genome‐wide methylation analysis of the mammary glands, comparing control versus LPS groups. (a) Whole mount staining of the mammary gland. (b) The grayscale image of the mammary gland using rgb2gray. (c) Meijering filter to enhance ridge‐like structures. (d) The skeletonized image of mammary ducts. (e) Tagged skeleton indicates the processed image by AnalyzeSkeleton. Blue box indicates the processes performed in python, and the red box indicates the processes performed in ImageJ software. (f) Depiction of DNA methylation across the genome. The outmost ring indicates the chromosome number 1 to 22, including X and Y chromosomes. Each dot on the plot marks a distinct genomic location with altered methylation. The blue dots represent hypermethylation and the red dots represent hypomethylation. (g) Illustration of the genomic regulatory region and CpG regulatory region of the DMRs, where the column with an asterisk represents significant differentially methylated regions (DMRs). The labels “hypo^∗^” and “hyper^∗^” indicate significant hypomethylation and hypermethylation, respectively. The absence of an asterisk signifies all hypo/hyper positions, regardless of their statistical significance in differential methylation. Additionally, “MM280” represents the overall distribution of the array. (h) Depiction of the KEGG pathway enrichment analyses of hypermethylated and hypomethylated regions, respectively. S.P. refers to signaling pathway. Panels (a–d) were processed using Python (v 3.9) with scikit‐image (v 0.16.0) and NumPy (v 1.26.2) libraries; panel (e) was processed using ImageJ software (v 1.54h) with the AnalyzeSkeleton plugin.

The image of whole mount staining of mammary glands was obtained using optical microscopy (Figure 1(a)). The image processing analysis was conducted using the Python programming language (v 3.9), in conjunction with ImageJ software (v 1.54h, https://imagej.net/ij/). The preliminary phase of the analysis was executed using scikit‐image (version 0.16.0) and NumPy (v 1.26.2) libraries. This phase involved the manipulation and detailed examination of whole‐mount staining images of mammary glands. The conversion of colored images to grayscale was achieved using the “rgb2gray” function (Figure 1(b)). Subsequently, the “meijering” function was employed to filter the images, effectively highlighting ridge‐like structures (Figure 1(c)). To refine the image quality, a morphological filter was applied to eliminate small objects, and Gaussian smoothing with a sigma value of 0.5 was utilized to diminish the noise of the images (Supporting File 2).

For the transformation of the grayscale images into binary format, Otsu’s method was performed. This technique calculates an optimal threshold value to separate the pixel classes into distinct groups. The process then progressed to skeletonization, where the binary image was converted into a skeletonized form, revealing the essential structure of mammary ducts (Figure 1(d)).

In the next phase of analysis, the AnalyzeSkeleton (https://fiji.sc/AnalyzeSkeleton) [36] plugin for ImageJ was used (Figure 1(e)). This plugin performs a comprehensive quantification of various morphological aspects, including the number of branches, junctions, and endpoints, measurement of junction and slab voxels, assessment of average and maximum branch lengths, and determination of triple and quadruple points.

### 2.9. Statistical Analysis

Statistical analysis for cytokine, miRNA expression, and mammary gland morphology data was conducted using GraphPad Prism (GraphPad Software, San Diego, CA, USA). The distribution of data was assessed using the Shapiro–Wilk test. For datasets that did not meet normality assumptions (e.g., cytokine concentrations), variables were log10‐transformed prior to statistical testing to achieve normality. Two‐way analysis of variance (ANOVA), followed by Tukey’s post hoc test, was performed to compare the means across the four experimental groups: (1) control (regular drinking water + PBS injection), (2) LPS (regular drinking water + LPS injection), (3) prebiotic/AHCC (AHCC in drinking water + PBS injection), and (4) prebiotic + LPS (AHCC in drinking water + LPS injection). Primary comparisons included control vs. LPS (to assess LPS effects), LPS vs. prebiotic + LPS (to evaluate AHCC mitigation), control vs. prebiotic (to assess AHCC alone), and prebiotic vs. prebiotic + LPS (for consistency). For multiplex cytokine data and miRNA expression profiling, multiple testing was addressed by adjusting *p* values using the false discovery rate (FDR) method to control for type I errors across comparisons. The results are presented as mean ± SEM, and a *p* value < 0.05 indicates a statistically significant difference between groups.

## 3. Results

### 3.1. Long‐Term Effects of LPS Exposure on DNA Methylation and Potential Impact of AHCC Treatment

Comparison of control and AHCC groups revealed no statistically significant DMRs (Supporting File 1, Figure S2). In the control versus LPS comparison, 85.8% of DMRs were hypomethylated and 14.1% were hypermethylated (Supporting Table 1). Global DNA methylation patterns across all chromosomes are shown in Figure 1(f). Hypomethylated DMRs were more frequent at promoters, 1–5 kb upstream of transcriptional start sites (TSSs), and exonic regions (Figure 1(g)). CpG density plots indicated a smaller proportion of hypermethylated regions in CpG islands, shelves, and shores, with a larger proportion of hypomethylated regions in these areas (Figure 1(g)). Hypermethylated DMRs were associated with PI3K‐Akt (4 DMRs), MAPK, Hippo, and Rap1 signaling pathways (3 DMRs each). Hypomethylated DMRs were associated with cAMP (18 DMRs), PI3K‐Akt (16 DMRs), Rap1 (14 DMRs), MAPK (14 DMRs), Ras signaling pathways (11 DMRs), cytokine–cytokine receptor interaction (19 DMRs), microRNAs in cancer (13 DMRs), and proteoglycan in cancer (12 DMRs) (Figure 1(h), Supporting Table 2).

In the LPS versus AHCC + LPS comparison, 29.9% of DMRs were hypomethylated and 70.1% were hypermethylated (Supporting Table 3). Global DNA methylation patterns across all chromosomes are shown in Figure 2(a). Hypermethylated DMRs were more frequent at promoters, 1–5 kb upstream of TSS, and exonic regions compared to hypomethylated DMRs (Figure 2(b)). CpG density plots indicated a smaller proportion of hypermethylated regions in CpG islands, shelves, and shores, with a larger proportion of hypomethylated regions in these areas (Figure 2(b)). Hypermethylated DMRs were associated with T‐cell receptor (6 DMRs), Ras (8 DMRs), NOD‐like receptor (6 DMRs), Rap1 (6 DMRs), cAMP (6 DMRs), MAPK (8 DMRs), PI3K‐Akt signaling pathways (7 DMRs), pathways in cancer (13 DMRs), microRNAs in cancer (8 DMRs), and cytokine–cytokine receptor interaction (8 DMRs). Hypomethylated DMRs were associated with tight junction (6 DMRs), Rap1 (6 DMRs), MAPK (6 DMRs), PI3K‐Akt signaling pathway (5 DMRs), and pathways in cancer (9 DMRs) (Figure 2(c), Supporting Table 4).

FIGURE 2Genome‐wide methylation analysis of the mammary glands, comparing LPS versus AHCC + LPS groups. (a) Depiction of DNA methylation patterns across the genome. The outmost ring indicates the chromosome numbers 1 to 22, including X and Y chromosomes. Each dot on the plot marks a distinct genomic location with altered methylation. The blue dots represent hypermethylation and the red dots represent hypomethylation. (b) Illustration of the genomic regulatory region and CpG regulatory region of the DMRs, where the column with an asterisk represents significant differentially methylated regions (DMRs). The labels “hypo^∗^” and “hyper^∗^” indicate significant hypomethylation and hypermethylation, respectively. The absence of an asterisk signifies all hypo/hyper positions, regardless of their statistical significance in differential methylation. Additionally, “MM280” represents the overall distribution of the array. (c) Depiction of the KEGG pathway enrichment analyses of hypermethylated and hypomethylated regions, respectively. S.P. refers to signaling pathway. (d) Circular plot indicating the role of 14 genes in critical signaling pathways (S.P.). (e) The methylation level of 14 genes, *Ntng1*, *Vav3*, *Ppp3ca*, *Pdgfa*, *Pdgfc*, *Jag2*, *Nr4a1*, *Hras*, *Ksr1*, *Camk2g*, *Mybl2*, *Il2rb*, *Il17b*, and *Il17rb*, that were hypermethylated in AHCC + LPS and hypomethylated in LPS groups in mammary glands of adult mice 4 weeks after injection of a single dose of LPS at puberty.

**Figure:**
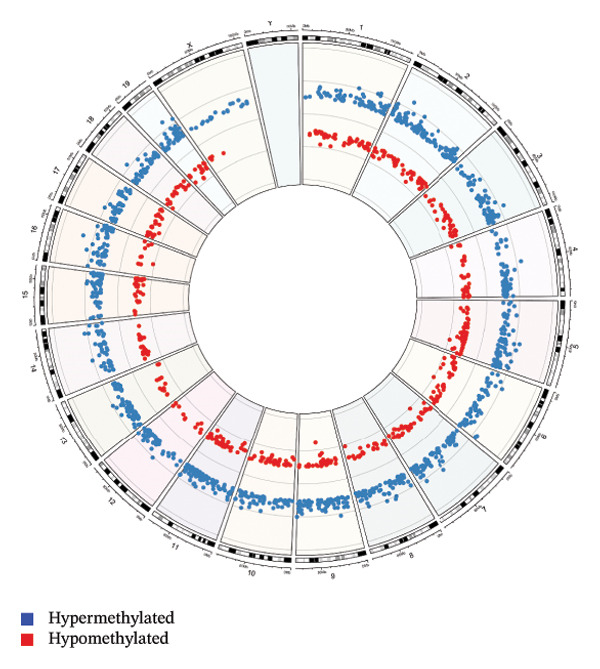
(a)

**Figure:**
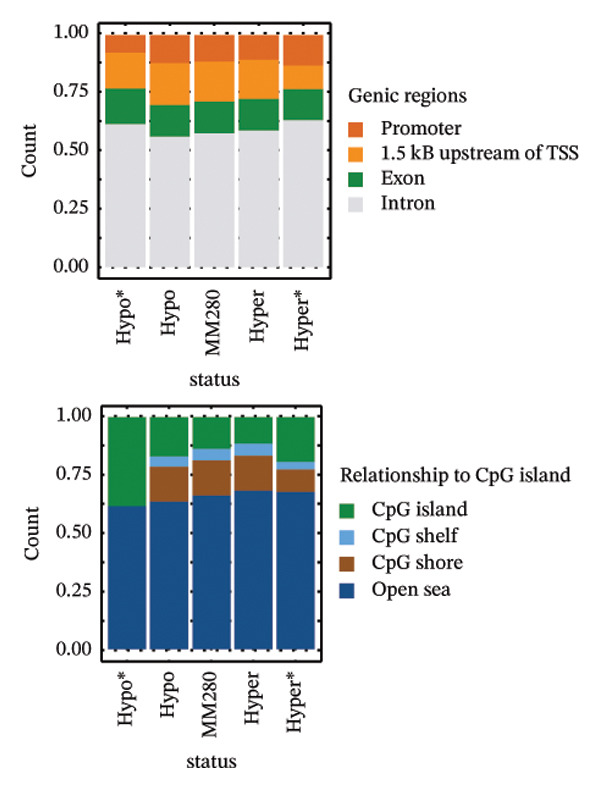
(b)

**Figure:**
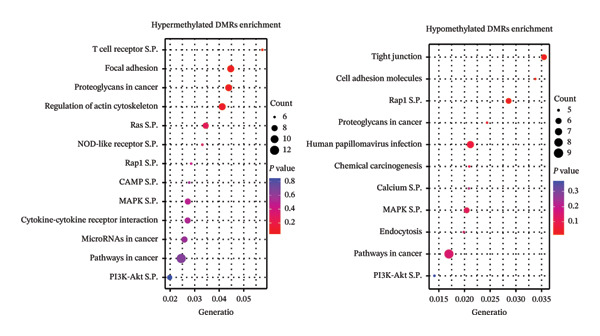
(c)

**Figure:**
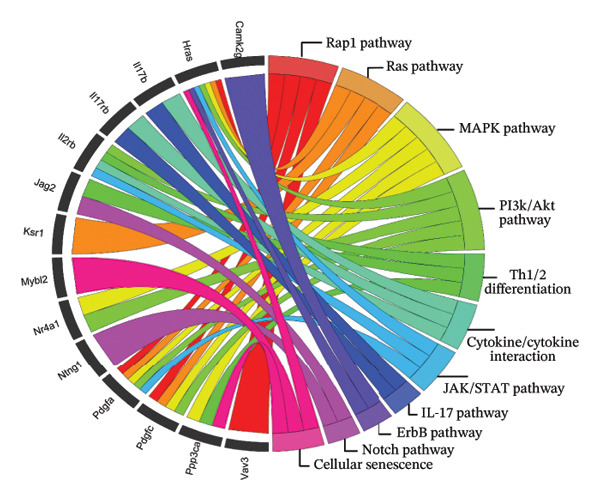
(d)

**Figure:**
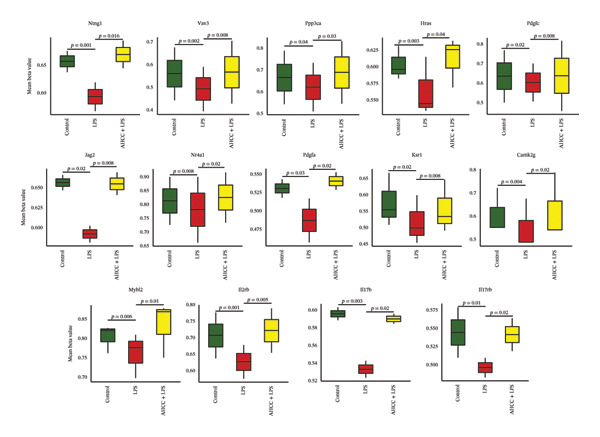
(e)

Overlapping analysis of genes differentially methylated in control vs. LPS and LPS vs. AHCC + LPS identified 252 shared genes, of which 14 were associated with signaling pathways including Ras, Rap1, MAPK, PI3K‐Akt, Th1 and Th2 cell differentiation, cytokine–cytokine receptor interaction, JAK‐STAT, and IL‐17 (Figure 2(d)). These genes included *Ntng1*, *Vav3*, *Ppp3ca*, *Pdgfa*, *Pdgfc*, *Jag2*, *Nr4a1*, *Hras*, *Ksr1*, *Camk2g*, *Mybl2*, *Il2rb*, *Il17b*, and *Il17rb* (Figure 2(e)). Additionally, 28 genes were hypermethylated in LPS vs. control but hypomethylated in AHCC + LPS vs. LPS. Among these, seven were involved in cancer development or tumor suppression: Rhobtb1, Dmtn, Zdhhc1, and Adarb1 (tumor suppressors) and Asph, Emp1, and Tmcc3 (tumorigenesis and metastasis inducers) (Supporting File 1, Figure S3).

### 3.2. Long‐Term Effects of LPS Exposure on Pro‐Inflammatory Cytokines and Immunomodulatory Effect of AHCC Treatment

Results showed that although IL‐1β levels were slightly decreased in the LPS group (∼1.1‐fold decrease) compared to the control group, this difference was not statistically significant. Interestingly, IL‐1β levels were significantly higher in the LPS group (∼2.3‐fold increase) than in the AHCC + LPS group. Although IL‐1β levels were lower in the AHCC (∼1.7‐fold decrease) and AHCC + LPS (∼2.5‐fold decrease) groups compared to the control group, these differences were not statistically significant.

The levels of IL‐6 were significantly decreased in the LPS (∼3.9‐fold decrease, *p* < 0.01), AHCC (∼3.7‐fold decrease, adjusted *p* < 0.01), and AHCC + LPS (∼3.4‐fold decrease, adjusted *p* < 0.01) groups compared to the control group. However, no significant differences were observed between the LPS and AHCC + LPS groups. This observation may be attributed to the transient nature of IL‐6 expression, which typically peaks early during acute inflammatory responses.

Regarding IL‐10, a tendency toward an increase in the LPS group (∼1.1‐fold increase) compared to the control group was observed; however, this difference was not statistically significant (adjusted *p* > 0.05). Interestingly, IL‐10 levels were significantly lower in the AHCC group (∼1.4‐fold decrease vs. LPS, adjusted *p* < 0.05) compared to the LPS group, whereas the difference versus the AHCC + LPS group (∼1.7‐fold decrease, adjusted *p* > 0.05) was not significant. No significant differences were observed between the LPS and AHCC + LPS groups.

Additionally, while IL‐17A levels were increased in all treatment groups relative to the control, the only statistically significant difference was observed between the control and LPS groups (∼2.0‐fold increase, adjusted *p* < 0.05). Moreover, IL‐17A levels were higher in the LPS group (∼1.7‐fold increase, adjusted *p* > 0.05) compared to the AHCC + LPS group, though this difference was not statistically significant.

IL‐17F levels were significantly higher in the LPS group (∼1.5‐fold increase, adjusted *p* < 0.05) compared to the control group. Interestingly, IL‐17F levels were significantly lower in the AHCC group (∼1.7‐fold decrease, adjusted *p* < 0.01) compared to the LPS group. Moreover, IL‐17F levels were lower in the AHCC + LPS group (∼1.2‐fold decrease) compared to the LPS group; however, this difference was not statistically significant (adjusted *p* > 0.05).

Finally, IL‐23 levels were lower in the AHCC group (∼2.0‐fold decrease vs. control, adjusted *p* < 0.05; ∼1.2‐fold decrease vs. LPS; ∼1.3‐fold decrease vs. AHCC + LPS) compared to the control, LPS, and AHCC + LPS groups. Although there was a trend toward an increase in IL‐23 levels in the LPS (∼1.2‐fold increase vs. AHCC) and AHCC + LPS (∼1.3‐fold increase vs. AHCC) groups, no statistically significant differences were observed between these two groups (Figure 3(a)).

FIGURE 3Impact of pubertal LPS exposure and AHCC treatment on mammary gland cytokine concentrations and microRNA expression levels. (a) Concentrations of IL1β, IL‐6, IL‐10, IL‐17a, IL‐17f, and IL‐23 in the mammary glands of adult mice 4 weeks after the injection of a single dose of LPS at puberty. Two‐way ANOVA and Tukey’s post hoc tests were used to compare groups. (b) DNA methylation patterns of Dicer1 and Ago2, two pivotal genes in microRNA maturation, in the LPS group compared to the control. Exact adjusted *p* values are shown for pairwise comparisons in panel (b) due to the limited number of comparisons and specific focus on methylation differences; for panels (a, c, and d), asterisks represent significance levels based on adjusted *p* values from Tukey’s test to account for multiple comparisons. (c) The relative expression of candidate tumor suppressor microRNAs: let‐7a, let‐7c, miR‐34a, miR‐130a, miR‐146a, miR‐184, miR‐200c, miR‐211, and miR‐219 in the mammary glands of adult mice 4 weeks after a single LPS injection at puberty. Two‐way ANOVA and Tukey’s post hoc tests were used to compare groups. (d) The number of quadruple points in mammary glands across experimental groups. All values are expressed as mean ± SEM. ^∗^ *p* < 0.05, ^∗∗^ *p* < 0.01, ^∗∗∗^ *p* < 0.001, and ^∗∗∗∗^ *p* < 0.0001.

**Figure:**
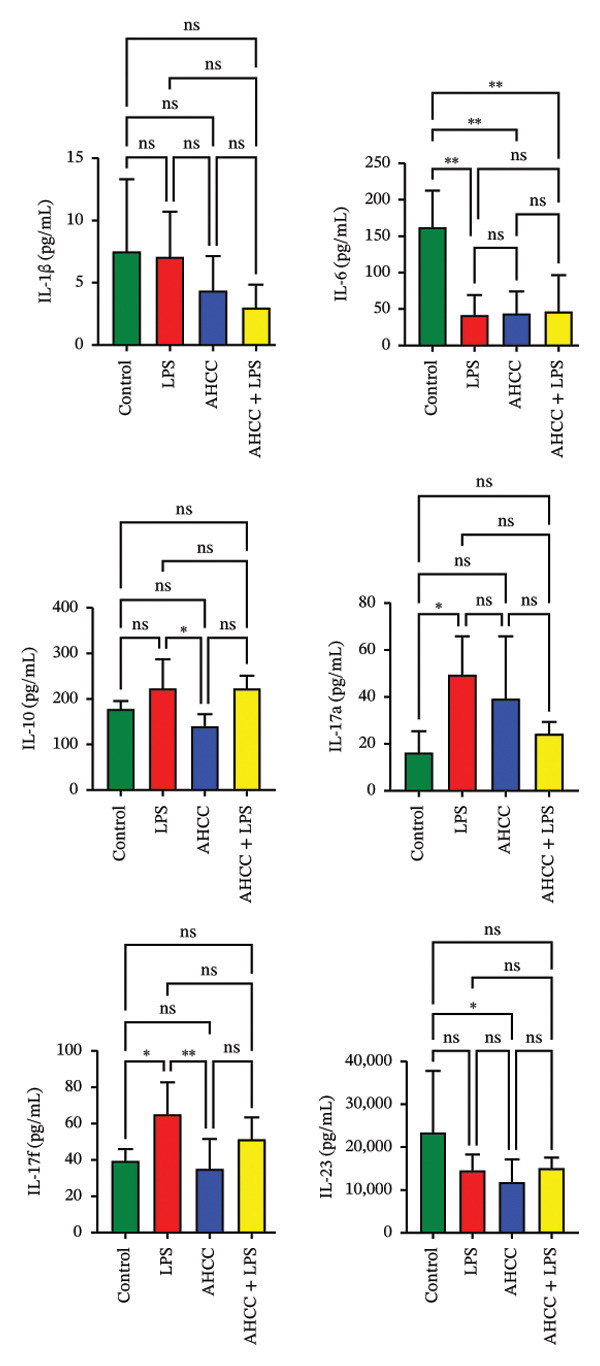
(a)

**Figure:**
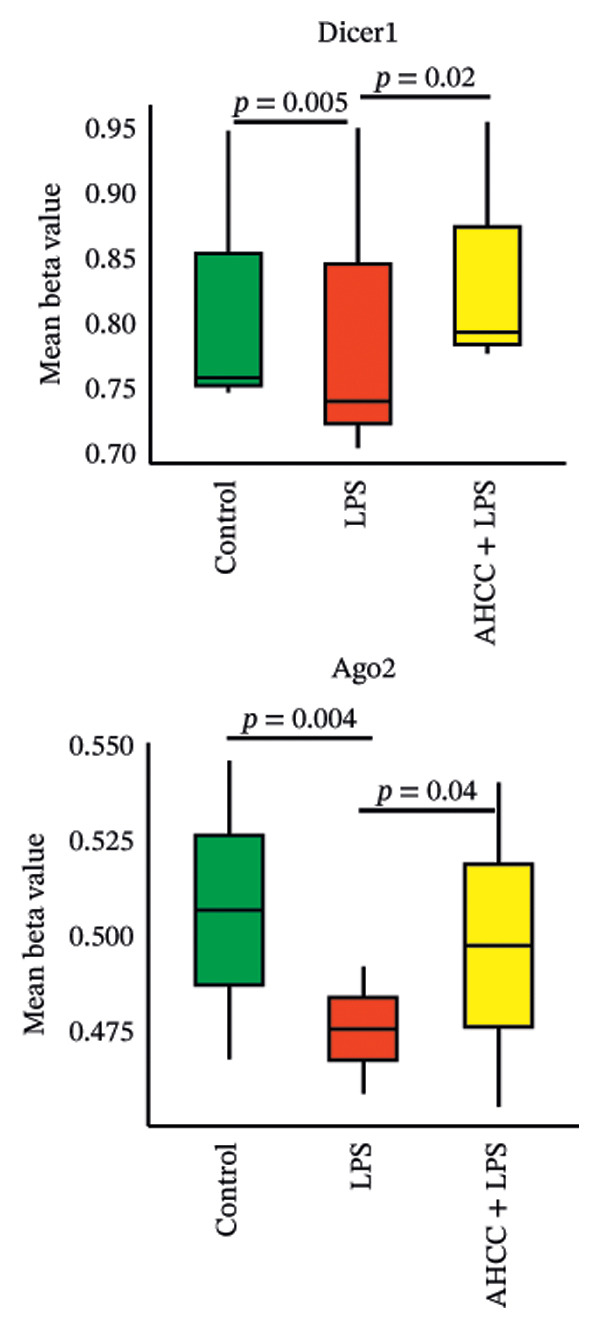
(b)

**Figure:**
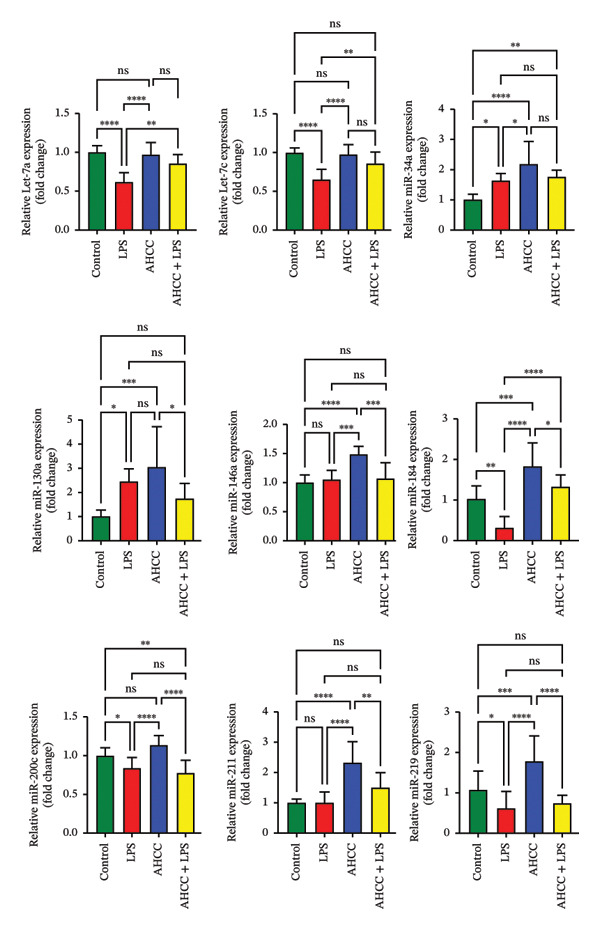
(c)

**Figure:**
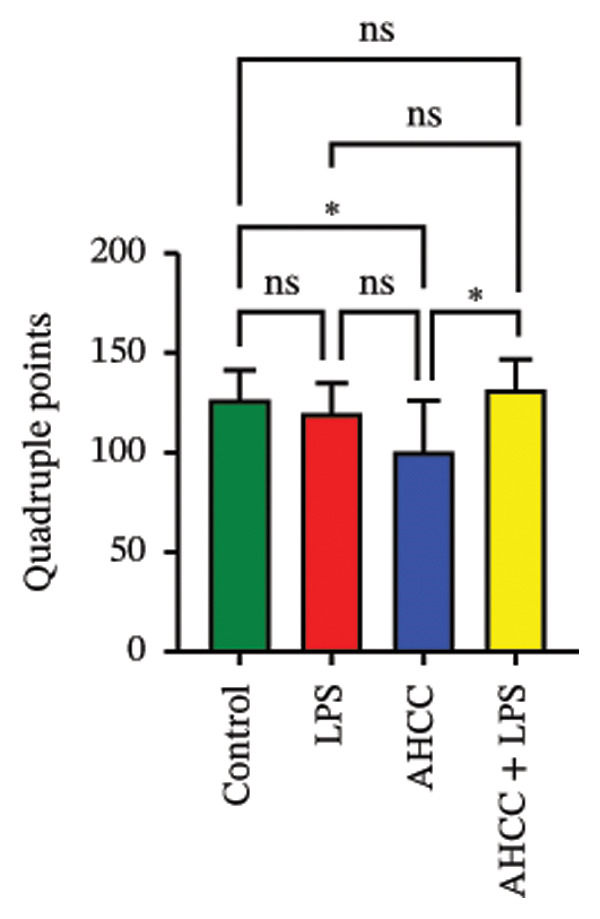
(d)

### 3.3. Long‐Term Effects of LPS Exposure on microRNAs and Mammary Gland Morphology and the Potential Impact of AHCC Treatment

DNA methylation analysis revealed that Dicer1 and the intronic region of Ago2, both essential for microRNA maturation, were significantly hypomethylated in the LPS group compared to the control group (Dicer1: mean beta value 0.85 in control vs. 0.75 in LPS, difference −0.10, crude *p* = 0.005; Ago2: 0.52 in control vs. 0.48 in LPS, difference −0.04, crude *p* = 0.04) (Figure 3(b)).

The findings revealed a statistically significant downregulation of let‐7a (∼1.6‐fold decrease) and let‐7c (∼1.5‐fold decrease) in the LPS group (adjusted *p* < 0.01), while no significant changes were observed in the AHCC group vs. control. Furthermore, the expression of let‐7a/c (∼1.2‐fold decrease) was slightly decreased in the AHCC + LPS group compared to the control group; however, these differences were not statistically significant (adjusted *p* > 0.05). Notably, the expressions of let‐7a (∼1.4‐fold increase) and let‐7c (∼1.3‐fold increase) were significantly higher in the AHCC + LPS group compared to the LPS group (adjusted *p* < 0.01).

In this study, an upregulation of miR‐34a was observed in all experimental groups compared to the control group (LPS: ∼1.6‐fold increase, adjusted *p* < 0.05; AHCC: ∼2.2‐fold increase, adjusted *p* < 0.01; AHCC + LPS: ∼1.8‐fold increase, adjusted *p* < 0.01). miR‐34a expression was ∼1.4‐fold higher in the AHCC group compared to the LPS group. Likewise, a similar trend was observed for miR‐130a, another well‐known tumor suppressor microRNA, which exhibited an upregulation in all experimental groups compared to the control group (LPS: ∼2.4‐fold increase, adjusted *p* < 0.05; AHCC: ∼3.0‐fold increase, adjusted *p* < 0.01; AHCC + LPS: ∼1.7‐fold increase, adjusted *p* < 0.05).

In contrast, no significant change was observed in the expression of miR‐146a in the LPS and AHCC + LPS groups compared to the control group, indicating that LPS exposure did not have a significant effect on the expression of this microRNA in the mammary glands. However, it is noteworthy that the expression level of miR‐146a was found to be significantly increased in the AHCC group (∼1.5‐fold increase vs. control; ∼1.4‐fold increase vs. LPS; ∼1.4‐fold increase vs. AHCC + LPS) compared to all other experimental groups (adjusted *p* < 0.01).

The results demonstrate that miR‐184 expression was significantly decreased in the LPS group (∼1.8‐fold decrease, adjusted *p* < 0.01) compared to the control group. Additionally, there was a slight increase in miR‐184 expression in the AHCC + LPS group (∼1.3‐fold increase, adjusted *p* > 0.05) compared to the control group, and miR‐184 expression was higher in the AHCC + LPS group (∼2‐fold increase, adjusted *p* < 0.01) compared to the LPS group. Moreover, the findings indicate that AHCC treatment increased miR‐184 expression compared to the control group (∼1.8‐fold increase vs. control, adjusted *p* < 0.01).

In the study, a slight downregulation of miR‐200c was observed in both the LPS (∼1.2‐fold decrease, adjusted *p* < 0.05) and AHCC + LPS (∼1.3‐fold decrease, adjusted *p* < 0.01) groups compared to the control group. On the other hand, the expression of miR‐200c was found to be slightly higher in the AHCC group (∼1.1‐fold increase) compared to the control group, although this difference was not statistically significant (adjusted *p* > 0.05).

The expression of miR‐211 was found to be unaltered in the LPS group when compared to the control group. However, a significant increase was observed in the AHCC (∼2.3‐fold increase, adjusted *p* < 0.01) and AHCC + LPS (∼1.5‐fold increase, adjusted *p* < 0.01) groups in comparison to the control group. Moreover, the expression level of miR‐211 was observed to be higher in the AHCC + LPS group (∼1.5‐fold increase) compared to the LPS group, although this difference was not statistically significant. These results suggest that AHCC treatment may positively regulate the expression of miR‐211 in mammary glands and that LPS exposure alone may not affect its expression.

In addition, the research findings revealed that miR‐219 was significantly downregulated in response to LPS exposure (∼1.7‐fold decrease, adjusted *p* < 0.05), whereas it was significantly upregulated following AHCC treatment (∼1.7‐fold increase, adjusted *p* < 0.01). Additionally, data showed that the expression level of miR‐219 decreased in the AHCC + LPS group (∼1.4‐fold decrease) compared to the control group; however, no significant difference was observed between the LPS and AHCC + LPS groups. These results suggest that LPS exposure can downregulate critical tumor suppressor microRNAs, such as let‐7a, let‐7c, miR‐184, and miR‐219, while AHCC treatment may mitigate this effect and restore their expression levels (Figure 3(c)).

Mammary gland morphological parameters, including the number of branches, junctions, endpoints, junction voxels, slab voxels, average branch length, maximum branch length, and triple points, showed no significant differences across groups (Supporting File 1, Figure S4). The number of quadruple points was significantly lower in the AHCC group compared to the control and AHCC + LPS groups (Figure 3(d)).

## 4. Discussion

In this study, we observed no statistically significant DMRs between control and AHCC groups. In the control versus LPS comparison, 85.8% of DMRs were hypomethylated and 14.1% were hypermethylated, with hypomethylated DMRs enriched at promoters, 1–5 kb upstream of TSSs, and exonic regions, as well as in CpG islands, shelves, and shores. These DMRs were associated with pathways including cAMP, PI3K‐Akt, Rap1, MAPK, Ras, cytokine–cytokine receptor interaction, microRNAs in cancer, and proteoglycans in cancer. In the LPS versus AHCC + LPS comparison, 29.9% of DMRs were hypomethylated and 70.1% were hypermethylated, with hypermethylated DMRs similarly enriched in promoter and exonic regions but showing associations with T‐cell receptor, Ras, NOD‐like receptor, Rap1, cAMP, MAPK, PI3K‐Akt signaling, pathways in cancer, microRNAs in cancer, and cytokine–cytokine receptor interaction. Overlapping analysis identified 252 shared genes, including 14 involved in signaling pathways such as Ras, Rap1, MAPK, PI3K‐Akt, Th1/Th2 cell differentiation, cytokine–cytokine receptor interaction, JAK‐STAT, and IL‐17 (e.g., *Ntng1, Vav3, Ppp3ca, Pdgfa, Pdgfc, Jag2, Nr4a1, Hras, Ksr1, Camk2g*, *Mybl2, Il2rb, Il17b, and Il17rb*). Additionally, LPS exposure led to altered cytokine levels (e.g., increased IL‐17A and IL‐17F and decreased IL‐6) and downregulation of tumor suppressor microRNAs (e.g., let‐7a, let‐7c, miR‐184, and miR‐219), which were partially mitigated by AHCC treatment. Mammary gland morphology showed no major differences across groups, except for reduced quadruple points in the AHCC group.

Dietary intervention with prebiotics has been proven to counteract the negative effects of gut microbiota dysbiosis [37, 38]. Building on this, our study aimed to examine whether AHCC (a mushroom‐derived supplement with immunomodulatory properties) could alleviate prolonged inflammatory immune responses in mice exposed to LPS during puberty, focusing on mammary glands and analyzing regulatory signaling pathways and epigenetic mechanisms involved.

Microbiome dysbiosis increases circulating LPS levels, which in turn exacerbate gut dysbiosis. This cycle of dysbiosis and elevated LPS triggers systemic effects including immune and epigenetic disruptions, such as altered DNA methylation patterns and cytokine expression dysregulation [38]. Particularly during puberty, such disruptions are linked to an increased risk of pre‐carcinogenic lesions in mammary glands [39]. Circulating LPS impacts gene expression in these glands primarily through the activation of the NF‐kB signaling pathway, leading to proinflammatory cytokine production and potentially abnormal gland growth [40, 41]. These changes are precursors to conditions like mastitis, influenced by elevated IL‐1β and IL‐6 levels [42]. Moreover, LPS‐induced inflammation can disrupt DNA methyltransferases, leading to cancer‐like DNA methylation patterns [43]. LPS also promotes Th17 cell differentiation and associated cytokine expression, contributing to chronic inflammation [44]. Furthermore, our findings underscore the dependence of miRNA expression on gut microbiota, with miRNAs at intestinal epithelial cells activating pathways and modulating gene expression [45]. Several studies corroborate the impact of LPS and inflammatory cytokines on miRNA dynamics in various tissues [46]. In line with this, our results demonstrated that AHCC treatment reversed LPS‐induced hypomethylation in key genes and pathways, restored certain microRNA expressions, and modulated cytokine profiles in mouse mammary glands.

In this study, we investigated the long‐term effects of LPS exposure on DNA methylation and the potential impact of AHCC treatment on the DNA methylation profile of mammary glands. Our results indicated significant methylation changes: the control vs. LPS group exhibited 85.8% hypomethylation and 14.1% hypermethylation, whereas the LPS vs. AHCC + LPS group showed 29.9% hypomethylation and 70.1% hypermethylation. Among the genes analyzed, 252 were hypomethylated in the LPS group compared to the control and hypermethylated in the AHCC + LPS group compared to LPS. Notably, 14 genes, including *Ntng1*, *Vav3*, *Ppp3ca*, *Pdgfa*, *Pdgfc*, *Jag2*, *Nr4a1*, *Hras*, *Ksr1*, *Camk2g*, *Mybl2*, *Il2rb*, *Il17b*, and *Il17rb*, played significant roles in key signaling pathways like cytokine–cytokine receptor interaction, JAK‐STAT, IL17 signaling, and microRNA processes.

For instance, *Netrin G1 (Ntng1)* and *Jagged2 (Jag2)* are essential in cell survival signaling and linked to colorectal and breast cancer through the Notch pathway [47, 48]. *Vav3* influences hormone‐dependent breast cancers by modulating estrogen receptor alpha transcription, affecting endocrine therapy resistance [49, 50]. *Ppp3ca* affects calcium‐dependent signaling and immune regulation, impacting breast cancer invasion [51, 52]. The platelet‐derived growth factors *Pdgfa* and *Pdgfc* are implicated in EMT and metastasis in gastrointestinal and breast cancers [53, 54]. *Nr4a1*, a member of the nuclear receptor subfamily, interacts with several key proteins including mTOR and HIF‐1α, contributing to tumor progression and micrometastasis induction [55, 56]. *Hras* and *Ksr1* regulate the Ras pathway, affecting cell growth and survival, with contrasting roles in cancer aggressiveness and patient survival [57]. *Camk2g, Mybl2,* and *Il2rb* influence ovarian cancer, breast cancer subtype expression, and cytokine interactions, respectively, affecting tumor aggressiveness and survival [58–60]. *I17b* and its receptor *Il17rb* play roles in inflammatory responses, promoting cancer cell growth and invasion [61]. These associations highlight how LPS‐induced hypomethylation may influence cancer‐related pathways in this model, with AHCC treatment leading to hypermethylation that could modulate these effects.

Our results also indicated that AHCC treatment reduced methylation in tumor suppressor genes like *Rhobtb1*, *Dmtn*, *Zdhhc1*, and *Adarb1*, which were hypermethylated under LPS exposure [62–65]. Conversely, genes like *Asph*, *Emp1*, and *Tmcc3*, associated with tumor progression, showed consistent methylation patterns. These observations demonstrate AHCC’s modulation of LPS‐induced epigenetic changes in mouse mammary glands.

Our study investigates the effects of DNA methylation on genes crucial for microRNA production and maturation. Notably, hypomethylation at the promoter of *Dicer1* and the intron of Ago2 was observed in the LPS group compared to the control. *Dicer1*, pivotal in microRNA maturation, plays a dual role in cancer dynamics—its loss is linked to tumorigenesis, while its overexpression is associated with poor outcomes in colorectal cancer. Argonaute 2 (Ago2), essential for the RISC protein complex, influences gene expression regulation, with its dysregulation impacting gene expression [66].

DNA methylation alterations due to LPS exposure resulted in both hypo‐ and hypermethylation of genes integral to signaling pathways such as cytokine–cytokine receptor interaction, Jak‐STAT, and IL17, highlighting inflammation’s role in modulating DNA methylation and affecting cellular processes. Previous research linked pubertal LPS exposure to altered gut microbiome compositions and increased proinflammatory cytokines, affecting mucosal integrity and potentially inducing abnormal mammary gland growth through NF‐κB activation [2, 67].

IL‐6 levels were unexpectedly low across all groups, contrary to typical LPS‐induced responses, which may be due to the timing of data collection, as IL‐6 expression is transient and peaks early in immune responses [68, 69]. Further investigations are needed to clarify the dynamics and regulation of IL‐6 under LPS and AHCC treatments.

Our research shows that LPS exposure induces a complex immune response in the mammary glands, characterized by elevated levels of both proinflammatory cytokines (IL‐1β, IL‐6, IL‐17A, IL‐17F, and IL‐23) and the anti‐inflammatory cytokine IL‐10. In particular, we noted higher levels of IL‐17A and IL‐17F in the LPS group compared to controls, with a slight, though not significant, increase in IL‐10 and IL‐23 levels. Interestingly, AHCC treatment in the AHCC + LPS group reduced the levels of IL‐17A and IL‐17F, suggesting a moderating effect of AHCC on LPS‐induced proinflammatory responses in this context. IL‐17, predominantly produced by Th17 cells, is known for its role in promoting immune cell recruitment and increasing proinflammatory cytokines and is linked to tumor progression in breast cancer via NF‐κB and MAPK pathways [70, 71]. While IL‐10 typically exhibits anti‐inflammatory properties, we observed a nonsignificant increase in the LPS group and significantly lower levels in the AHCC group compared to both LPS and AHCC + LPS groups. This indicates AHCC’s potential to modulate IL‐10 production under inflammatory conditions, which could influence the balance between proinflammatory and anti‐inflammatory signals [72, 73]. IL‐23 levels were marginally increased in the LPS and AHCC + LPS groups, though not significantly, and were noticeably reduced in the AHCC group. This reduction could reflect AHCC’s immunomodulatory effects, potentially inhibiting inflammation‐promoting mechanisms associated with IL‐23 in inflammatory diseases [74, 75]. These cytokine modulations by AHCC in our mouse model warrant further study to explore underlying mechanisms.

This study explores the impact of LPS exposure and AHCC treatment on the expression of tumor suppressor microRNAs, crucial for mammary gland biology, including let‐7a, let‐7c, miR‐184, and miR‐219. Our findings reveal a significant downregulation of these microRNAs in the LPS group, indicating LPS‐induced suppression that could disrupt key regulatory processes like cell proliferation, differentiation, and apoptosis in this model [20, 76]. Similarly, miR‐184, which regulates similar processes, was also downregulated, indicating a broader disruption caused by LPS exposure [77, 78]. Additionally, miR‐219’s downregulation in the LPS group contrasts its significant upregulation by AHCC treatment compared to the control, underscoring its potential role in neuroprotection and synaptic functions, which could extend to cellular processes in the mammary glands [79]. Interestingly, AHCC treatment did not significantly alter the expression levels of let‐7a, let‐7c, and miR‐184, suggesting its selective modulatory effects on microRNAs. This selective restoration of miR‐219 by AHCC indicates a specific pathway through which AHCC may counteract LPS‐induced disruptions. Further research is required to delineate the precise mechanisms by which AHCC influences miR‐219 expression and its broader implications for mammary gland health and disease prevention.

Our study observed notable differences in microRNA expression in response to LPS exposure and AHCC treatment. Unlike the downregulated let‐7a, let‐7c, miR‐184, and miR‐219, both miR‐34a and miR‐130a were significantly upregulated across all experimental groups compared to the control. miR‐34a, recognized for its tumor‐suppressive functions such as cell cycle arrest and apoptosis, showed increased levels suggesting a protective response against LPS‐induced damage [80]. This upregulation may reflect a cellular defense mechanism to mitigate the harmful effects of inflammation.

Similarly, the elevation of miR‐130a, involved in regulating cell proliferation and migration, particularly in breast cancer contexts, implies a potential safeguarding role in maintaining mammary gland integrity under inflammatory conditions [81]. Notably, the AHCC group exhibited higher levels of miR‐34a than the LPS group, indicating that AHCC might further boost this microRNA’s expression, potentially enhancing its protective effects.

Conversely, miR‐146a, known for its anti‐inflammatory role by targeting proinflammatory signaling pathways, did not vary significantly in the LPS and AHCC + LPS groups, suggesting that LPS exposure alone may not impact its expression in mammary glands [82]. However, its expression was significantly higher in the AHCC group, hinting at AHCC’s possible role in modulating this microRNA to exert anti‐inflammatory effects. Further investigation is needed to unravel the specific mechanisms through which miR‐130a and miR‐146a exert their effects and how AHCC may enhance these microRNAs’ expressions, contributing to its observed benefits in inflammatory conditions.

This study reveals the complexities of microRNA regulation in response to LPS exposure and AHCC treatment within mammary glands. We observed a slight downregulation of *miR-200c* in the LPS and AHCC + LPS groups, suggesting a potential disruption of epithelial–mesenchymal transition (EMT) and increased metastatic risk in breast cancer. However, AHCC treatment alone significantly upregulated *miR-200c* expression, indicating its possible role in maintaining EMT regulation and suppressing metastasis [83, 84]. Moreover, *miR-211* was significantly upregulated in both the AHCC and AHCC + LPS groups. Known for its involvement in melanocyte development and pigmentation, *miR-211*’s upregulation suggests AHCC may also modulate other critical aspects of mammary gland biology, potentially impacting pigmentation and cellular regulation [85].

Overall, these findings underscore the intricate interplay among LPS exposure, microRNA dynamics, and AHCC’s potential therapeutic effects in managing mammary gland health. AHCC appears to selectively modulate key microRNAs, potentially offering protective and anti‐inflammatory roles.

Following the evaluation of DNA methylation alterations, cytokine levels, and microRNA dysregulation, the morphological structure of mammary glands was assessed. This evaluation determined that neither LPS exposure during puberty nor AHCC treatment significantly altered the structure of mammary glands. However, a notable observation was the decreased number of quadruple intersections in the AHCC group compared to the control and AHCC + LPS groups.

This research is pioneering in its exploration of the links between inflammatory exposure during puberty and the development of a pre‐carcinogenic signature in adult mammary glands. Consistent with our previous work demonstrating LPS‐induced microRNA disruptions associated with breast cancer suppression [86], we observed significant changes in DNA methylation patterns following LPS exposure, with a predominance of hypomethylation akin to patterns seen in cancer cells—underscoring a potential carcinogenic risk. Concurrently, LPS exposure disrupted cytokine expression and altered the expression of inflammation‐associated microRNAs, affecting various signaling pathways. AHCC treatment showed promising results by partially restoring DNA methylation patterns and regulating key genes within cytokine–cytokine receptor interaction, JAK‐STAT, and IL‐17 signaling pathways, highlighting its potential to modulate inflammatory responses and restore immune processes in mammary glands affected by LPS. This indicates AHCC’s role as a modulator of epigenetic and inflammatory changes induced by LPS in this model. Future research is essential to further understand the mechanisms involved and the clinical relevance of AHCC in preventing dysfunction in mammary glands linked to inflammatory exposures and gut microbiome dysbiosis. This knowledge could lead to innovative strategies for the prevention and management of related mammary gland disorders.

### 4.1. Limitations

This study has several limitations. Sample sizes were relatively small (9 mice per group for cytokine and microRNA analyses; at least 5 per group for DNA methylation), potentially limiting statistical power to detect subtle effects. Experiments used a single mouse strain (BALB/c) and only female mice, restricting generalizability to other strains or sexes, as responses to LPS and AHCC may vary. Assessments occurred at a single time point (4 weeks post‐LPS), without longitudinal evaluation to track temporal changes in methylation, cytokines, or microRNAs. Systemic inflammation from LPS may confound mammary‐specific effects, with possible off‐target influences from other organs lacking controls. The DNA methylation analysis suggested epigenetic changes in genes for mammary development and breast cancer, alongside microRNA dysregulation in signaling pathways; however, mRNA levels for additional candidate genes or microRNA targets were not analyzed, which could further clarify AHCC’s role in reducing LPS‐induced inflammation. Finally, gut dysbiosis—a plausible mediator—was not directly measured (e.g., via microbiota profiling), so discussions of gut–mammary axis interactions and AHCC’s protective effects are speculative and warrant caution, especially for human clinical relevance. Future work with larger, diverse models and multitime‐point analyses could address these.

## 5. Conclusion

In summary, pubertal exposure to a single dose of LPS in mice led to predominant hypomethylation of DNA in mammary glands, enrichment in cancer‐related pathways (e.g., PI3K‐Akt, MAPK, and Ras), elevated proinflammatory cytokines (e.g., IL‐17A and IL‐17F), and downregulation of tumor suppressor microRNAs (e.g., let‐7a, let‐7c, miR‐184, and miR‐219), with minimal morphological changes. AHCC treatment partially reversed these effects, promoting hypermethylation in overlapping genes, modulating cytokine profiles (e.g., reducing IL‐1β and IL‐17 levels), and restoring select microRNA expressions (e.g., miR‐219), suggesting its immunomodulatory potential in this model.

These findings highlight how early‐life inflammatory insults may induce long‐term epigenetic and immune disruptions in mammary tissues, akin to pre‐carcinogenic signatures. While AHCC shows promise in mitigating such changes, clinical implications for inflammation‐related mammary disorders remain preliminary, particularly without direct assessment of gut microbiota involvement. Further research in diverse models and human cohorts is needed to validate these mechanisms and explore translational applications.

## Author Contributions

All authors contributed to the data collection and analysis process. The manuscript was drafted by Hamed Yasavoli‐Sharahi.

## Funding

This research was funded by an NSERC Collaborative Research and Development Grant (532223‐18), AHCC Research Association, and New Frontiers in Research Fund—Exploration (NFRF 2019‐01497) granted to C.M. and Z.H. Additionally, H.Y‐S. was the recipient of an admission scholarship from the Faculty of Graduate and Postgraduate Studies at the University of Ottawa.

## Disclosure

Please note that if an author is affiliated with the International Agency for Research on Cancer/World Health Organization, the opinions expressed in this article are solely their own and may not necessarily reflect the positions, policies, or viewpoints of the International Agency for Research on Cancer/World Health Organization. All authors reviewed the manuscript.

## Ethics Statement

All animal experiments were approved by the Animal Care Committee of the University of Ottawa (approval code: HSe‐3191, approved on July 13, 2022). Animal care and use complied with the Canadian Council on Animal Care guidelines and followed the principles outlined in the ARRIVE guidelines (https://arriveguidelines.org). This study did not involve human participants.

## Conflicts of Interest

The authors declare no conflicts of interest.

## Supporting Information

Additional supporting information can be found online in the Supporting Information section.

## Supporting information


**Supporting Information 1** Supporting File 1 includes the following figures: Figure S1: study design illustrating the experimental timeline, LPS/PBS administration, and dietary intervention. Thirty‐six mice were divided into control and AHCC groups, with LPS or PBS administered at puberty, resulting in four experimental groups (*n* = 9 per group). Figure S2: heatmap of DNA methylation levels in mammary gland tissue across treatment groups. CpG site annotations indicate genomic features such as CpG islands, shores, and genic regions. Figure S3: differential methylation patterns for seven genes associated with cancer development and tumor suppression (Rhobtb1, Dmtn, Zdhhc1, Adarb1, Asph, Emp1, and Tmcc3) across treatment groups. Figure S4: the measurement of mammary gland structure. Different criteria, including (a) number of branches, (b) junctions, (c) endpoints, measurement of (d) junction and (e) slab voxels, assessment of (f) average branch length, and determination of (g) triple points of mammary glands.

**Supporting Information 2** Supporting File 2 provides the Python code used for pre‐processing and analyzing whole‐mount stained mammary gland images. The code supports grayscale conversion, ridge enhancement (via the Meijering filter), and noise reduction, as described in the Image Processing section of the manuscript.

## Data Availability

The DNA methylation data generated in this study are available in the GEO database under accession code GSE290994. Additional datasets utilized and/or analyzed during this study can be provided by the corresponding author upon reasonable request.
