# Supplementary material for: Lentinula edodes Cultured Extract Intake at Puberty Mitigates Inflammatory Signals at the Mammary Glands by the Involvement of Epigenetic Mechanisms in BALB/c Mice
Source: Breast J. 2026 Mar 31;2026:2122220. doi: 10.1155/tbj/2122220 (PMC13140170; doi:10.1155/tbj/2122220)
Supplement: Supplementary file 1 — Supporting Information 1 Supporting File 1 includes the following figures: Figure S1: study design illustrating the experimental timeline, LPS/PBS administration, and dietary intervention. Thirty‐six mice were divided into control and AHCC groups, with LPS or PBS administered at puberty, resulting in four experimental groups (n = 9 per group). Figure S2: heatmap of DNA methylation levels in mammary gland tissue across treatment groups. CpG site annotations indicate genomic features such as CpG islands, shores, and genic regions. Figure S3: differential methylation patterns for seven genes associated with cancer development and tumor suppression (Rhobtb1, Dmtn, Zdhhc1, Adarb1, Asph, Emp1, and Tmcc3) across treatment groups. Figure S4: the measurement of mammary gland structure. Different criteria, including (a) number of branches, (b) junctions, (c) endpoints, measurement of (d) junction and (e) slab voxels, assessment of (f) average branch length, and determination of (g) triple points of mammary glands. [file TBJ-2026-2122220-s001.docx]

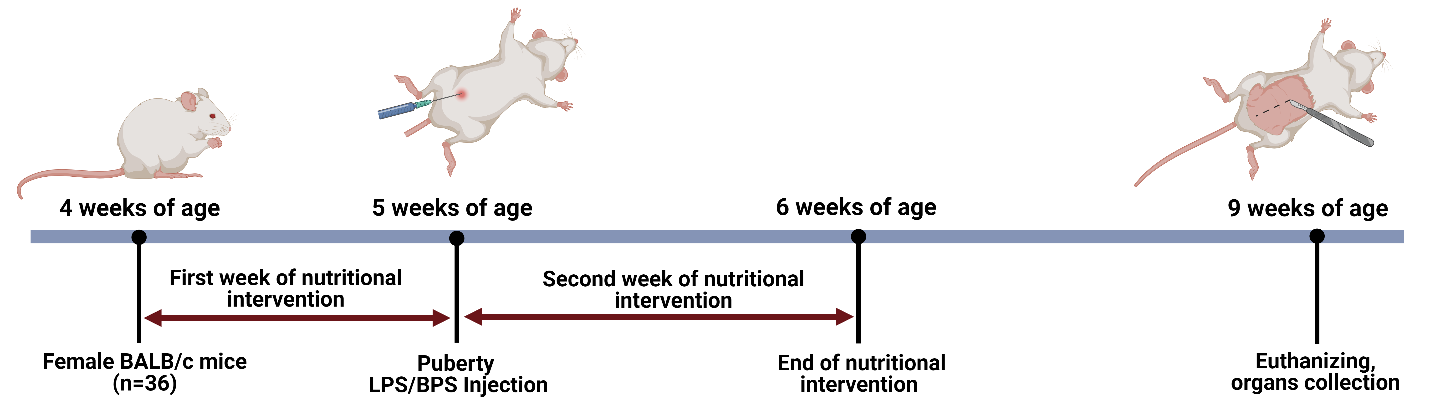


**Fig. S1. Study design showing the schematic of the research methodology, including the timeline of experiment, administration of LPS/PBS, and dietary intervention.** The study involves 36 mice, which are divided into two primary groups: control and AHCC. At the onset of puberty, 5 weeks of age, half of the mice within each group will receive LPS, while the remaining mice will receive PBS. This distribution results in formation of four experimental groups with 9 mice in each group.


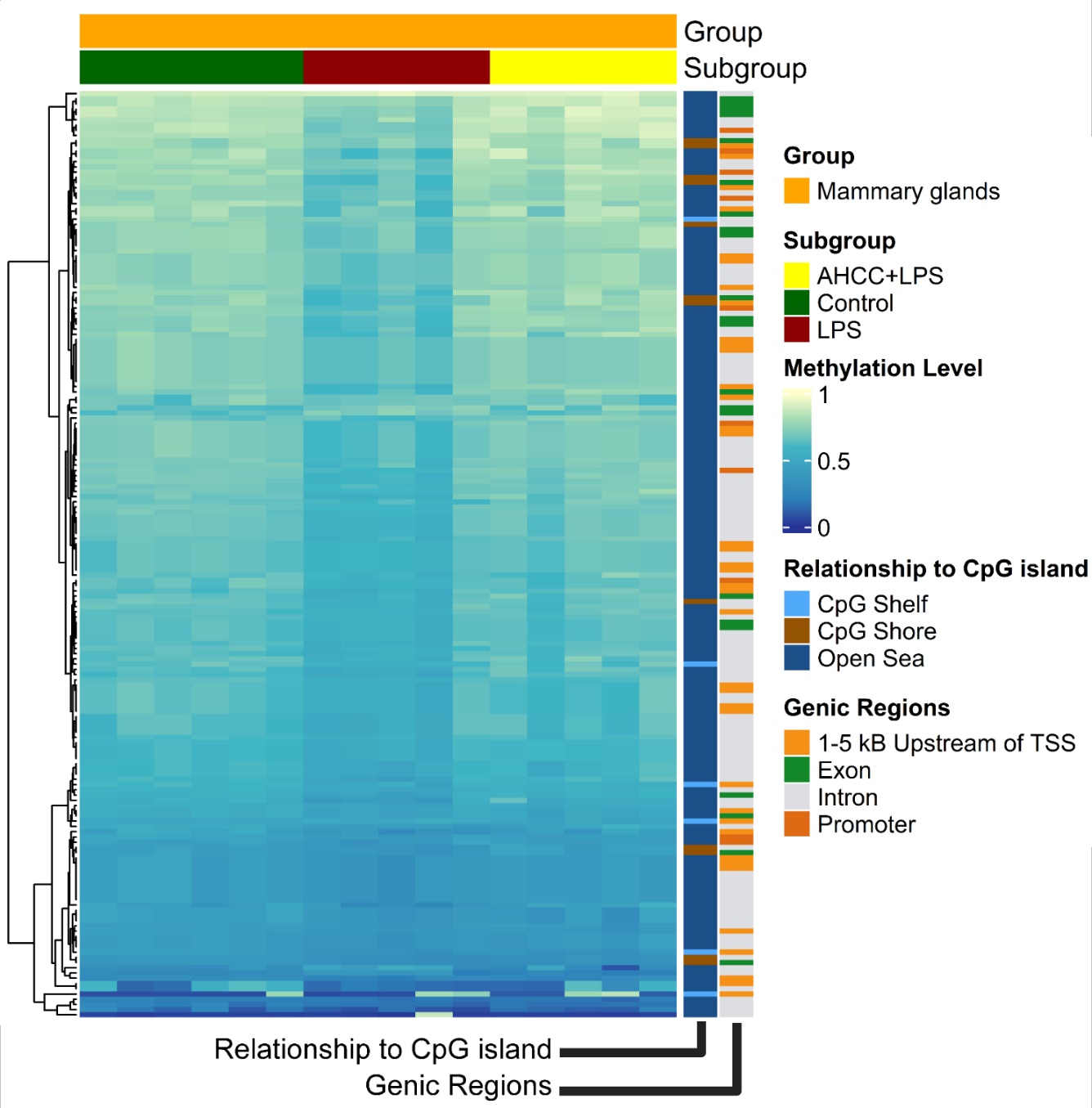


**Fig. S2. Heatmap of DNA methylation level across all the treatment groups.** The heatmap depict the methylation levels of various genomic regions in mammary glands across three groups: Control, LPS, and AHCC+LPS. Annotations on the right denote the relationship of CpG sites to CpG islands (CpG Shelf in orange, CpG Shore in blue, Open Sea in grey), and the specific genic regions affected (1-5 KB upstream of TSS in green, Exon in orange, Intron in blue, Promoter in grey), providing insights into the genomic context of methylation changes.


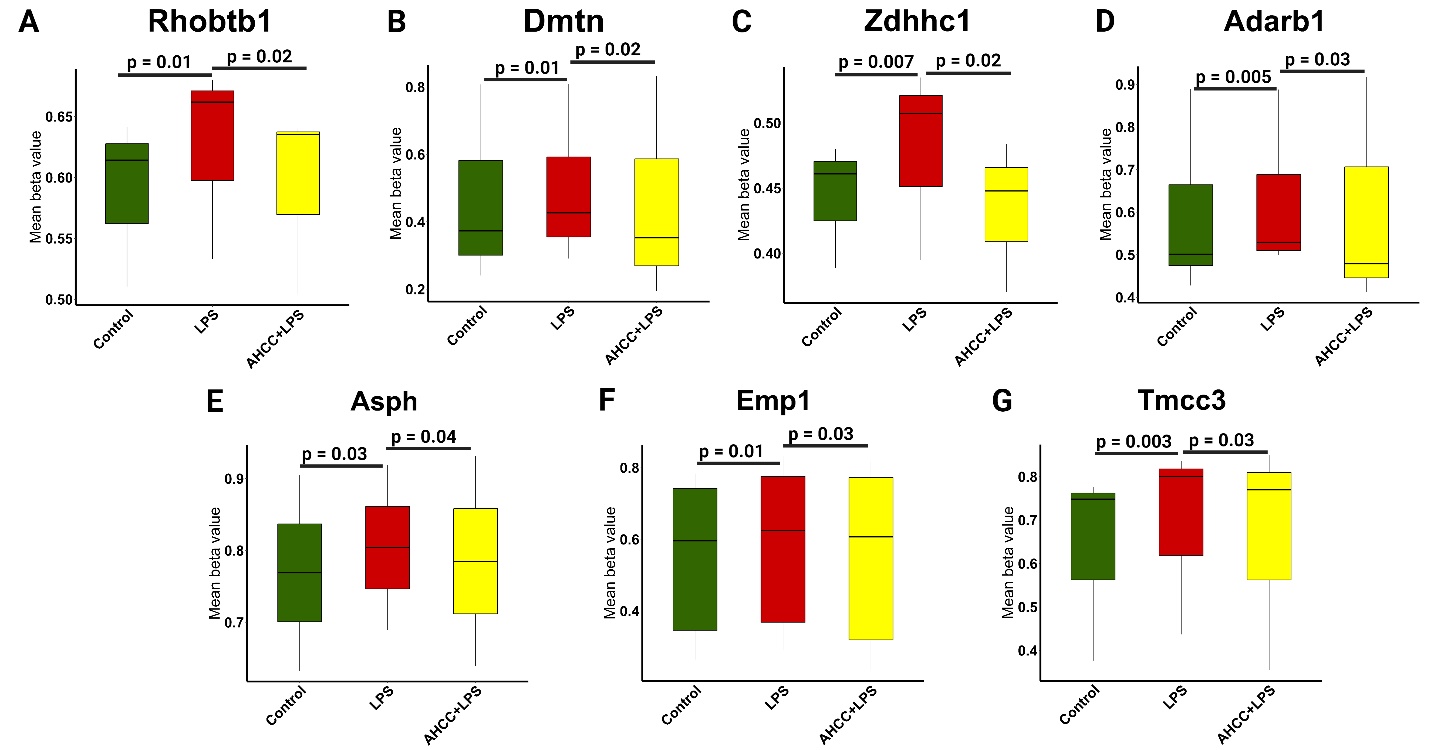


**Fig. S3. Differential methylation response to LPS and AHCC treatment.**  Mean beta values for seven genes associated with cancer development and tumor suppression across three treatment groups. The genes analyzed include **(a)** Rhobtb1, **(b)** Dmtn, **(c)** Zdhhc1, **(d)** Adarb1, **(e)** Asph, **(f)** Emp1, and **(g)** Tmcc3.


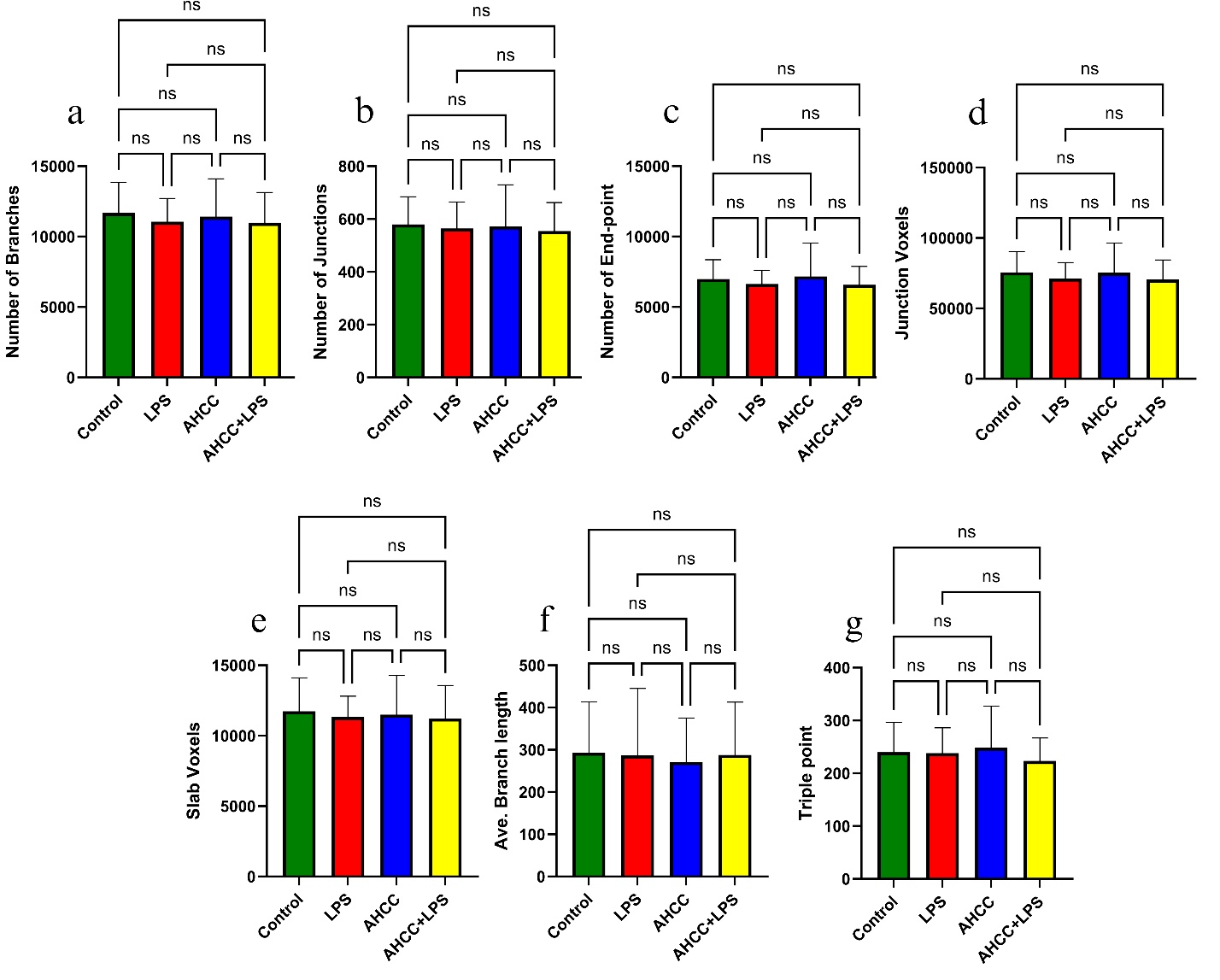


**Fig. S4.** **The Measurement of Mammary Gland Structure**. Different criteria, including **(a)** number of branches, **(b)** junctions, **(c)** endpoints, measurement of **(d)** junction and **(e)** slab voxels, assessment of **(f)** average branch length, and determination of **(g)** triple points of mammary glands were measured using the AnalyzeSkeleton plugin within ImageJ. Two-way ANOVA and Tukey’s post-hoc tests were used to compare groups. All values are expressed as mean ± SEM. *p < 0.05, **p < 0.01, ***p < 0.001, and ****p < 0.0001.
