# Supplementary material for: Lentinula edodes Cultured Extract Intake at Puberty Mitigates Inflammatory Signals at the Mammary Glands by the Involvement of Epigenetic Mechanisms in BALB/c Mice
Source: Breast J. 2026 Mar 31;2026:2122220. doi: 10.1155/tbj/2122220 (PMC13140170; doi:10.1155/tbj/2122220)
Supplement: Supplementary file 2 — Supporting Information 2 Supporting File 2 provides the Python code used for pre‐processing and analyzing whole‐mount stained mammary gland images. The code supports grayscale conversion, ridge enhancement (via the Meijering filter), and noise reduction, as described in the Image Processing section of the manuscript. [file TBJ-2026-2122220-s002.docx]

from skimage.filters import meijering

from skimage.restoration import (denoise_bilateral)

from skimage.color import rgb2gray

from skimage.util import img_as_float

import csv

from skimage import io, color, filters, util, morphology

from skimage.filters import gaussian

import numpy as np

import os

################

# SET DIRECTORIES

base_dir = r'D:\python mg analysis\all MG'

OriginalDir1 = os.path.join(base_dir, "Original Images")

GrayDir1 = os.path.join(base_dir, "Grayscale Images")

Destination1 = os.path.join(base_dir, "Processed Images")

HistogramDestination = os.path.join(base_dir, "Histograms")

csv_file = os.path.join(base_dir, "analysis_results.csv")

processed_image_path = os.path.join(Destination1, "my_processed_image.jpg")

# Ensure all directories exist

for directory in [OriginalDir1, GrayDir1, Destination1, HistogramDestination]:

if not os.path.exists(directory):

os.makedirs(directory)

#Check for Images in Original Directory

if not os.listdir(OriginalDir1):

print(f"No images found in {OriginalDir1}")

exit()

################

# CROP AND GRAYSCALE IMAGE

# Define the resolution (example: 100 pixels per mm)

resolution = 100 # Replace with your actual resolution

# Calculate the number of pixels to crop (1mm)

pixels_to_crop = resolution * 1 # 1mm

for filename in os.listdir(OriginalDir1):

try:

base_name = os.path.splitext(filename)[0]

if filename.endswith(".jpg"):

img = io.imread(os.path.join(OriginalDir1, filename), plugin='pil')

img2 = img.copy()

img_gray = rgb2gray(img2)

width = img_gray.shape[1]

height = img_gray.shape[0]

# Calculate new cropping boundaries

WL = pixels_to_crop

WR = width - pixels_to_crop

cropped = img_gray[0:height, WL:WR]

cropped = np.clip(cropped, 0, 1)

cropped_uint8 = (cropped * 255).astype(np.uint8)

new_filename = f"{base_name}.jpg"

F1 = os.path.join(GrayDir1, new_filename)

io.imsave(F1, cropped_uint8)

print(f"Processed {filename}")

else:

continue

except Exception as e:

print(f"Error processing {filename}: {e}")

##################

# SORT AND FILTER IMAGES

# Sort value of mean 89.99 optimized for present dataset. Adjust as needed.

for filename in os.listdir(GrayDir1):

if filename.endswith(".jpg"):

img_gray_cropped = io.imread(os.path.join(GrayDir1, filename),

plugin='pil')

base_name = os.path.splitext(filename)[0]

if np.mean(img_gray_cropped) > 10:

img_F = img_as_float(img_gray_cropped)

img_M = meijering(img_F)

img_filt = morphology.closing(img_M < 0.09)

img_r = morphology.remove_small_objects(~img_filt, min_size=100,

connectivity=10)

img_gray_cropped[~img_r] = 255

new_filename = f"{base_name}F.jpg"

F2 = os.path.join(Destination1, new_filename)

io.imsave(F2, img_gray_cropped)

else:

noisy = img_as_float(img_gray_cropped)

Bilateral = denoise_bilateral(noisy, sigma_color=0.1,

sigma_spatial=25,

multichannel=False)

new_filename = f"{base_name}B.jpg" # B for Bilateral

F3 = os.path.join(Destination1, new_filename)

io.imsave(F3, Bilateral)

else:

continue

##################

# Skeletonization

# Define the source and target directories

source_dir = r'D:\python mg analysis\all MG\Processed Images'

target_dir = r'D:\python mg analysis\all MG\Skeleton Images'

# Create the target directory if it doesn't exist

if not os.path.exists(target_dir):

os.makedirs(target_dir)

# Loop through each file in the source directory

for filename in os.listdir(source_dir):

print(f"Processing: {filename}") # Debug print

try:

if filename.lower().endswith(('.png', '.jpg', '.jpeg')):

image_path = os.path.join(source_dir, filename)

image = io.imread(image_path)

# Check if the image is RGBA, convert to RGB if it is

if image.ndim == 3 and image.shape[-1] == 4:

image = color.rgba2rgb(image)

# Convert the image to grayscale if it is RGB

if image.ndim == 3 and image.shape[-1] == 3:

gray_image = color.rgb2gray(image)

elif image.ndim == 2: # The image is already grayscale

gray_image = image

else:

print(f"Unsupported image format for {filename}, skipping.")

continue

# Smooth the image slightly to reduce noise

gray_image_smoothed = gaussian(gray_image, sigma=0.5)

# Threshold the image to get a binary image

thresh = filters.threshold_otsu(gray_image_smoothed)

binary_image = gray_image_smoothed > thresh

# Invert the binary image so the branches are True and the background is False

binary_image_inverted = util.invert(binary_image)

# Skeletonize the binary image

skeleton = morphology.skeletonize(binary_image_inverted)

# Define the path to save the skeleton image

skeleton_image_path = os.path.join(target_dir, f'skeleton_{filename}')

# Save the skeleton image

io.imsave(skeleton_image_path, skeleton.astype(np.uint8) * 255)

print(f"Saved: {skeleton_image_path}") # Confirm save

else:

print(f"Skipping non-image file: {filename}") # Non-image file

except Exception as e:

print(f"Error processing {filename}: {e}") # Print any errors
